# Improvement of the *Oryza sativa* Nipponbare reference genome using next generation sequence and optical map data

**DOI:** 10.1186/1939-8433-6-4

**Published:** 2013-02-06

**Authors:** Yoshihiro Kawahara, Melissa de la Bastide, John P Hamilton, Hiroyuki Kanamori, W Richard McCombie, Shu Ouyang, David C Schwartz, Tsuyoshi Tanaka, Jianzhong Wu, Shiguo Zhou, Kevin L Childs, Rebecca M Davidson, Haining Lin, Lina Quesada-Ocampo, Brieanne Vaillancourt, Hiroaki Sakai, Sung Shin Lee, Jungsok Kim, Hisataka Numa, Takeshi Itoh, C Robin Buell, Takashi Matsumoto

**Affiliations:** 1 Agrogenomics Research Center, National Institute of Agrobiological Sciences, 2-1-2 Kannondai, Tsukuba, Ibaraki, 305-8602 Japan; 2 Cold Spring Harbor Laboratory (CSHL), Cold Spring Harbor, NY 11723 USA; 3 Department of Plant Biology, Michigan State University, Plant Biology Laboratories, 612 Wilson Rd, East Lansing, MI 48824 USA; 4 Perkin Elmer, Room 4096, 8490 Progress Drive, Frederick, MD 21701 USA; 5 Laboratory for Molecular and Computational Genomics, University of Wisconsin-Madison, UW-Biotechnology Center, 425 Henry Mall, Madison, WI 53706 USA; 6 Integrated Center for Genes, Environment and Health, National Jewish Health, Denver, CO USA; 7 Dupont Pioneer, 7200 NW 62nd Ave, Johnston, IA 50131 USA

**Keywords:** *Oryza sativa*, Nipponbare, Unified rice reference genome, Pseudomolecules, Minimum tiling path, Optical mapping, Genome re-sequencing, Next-generation sequencing

## Abstract

**Background:**

Rice research has been enabled by access to the high quality reference genome sequence generated in 2005 by the International Rice Genome Sequencing Project (IRGSP). To further facilitate genomic-enabled research, we have updated and validated the genome assembly and sequence for the Nipponbare cultivar of *Oryza sativa* (*japonica* group).

**Results:**

The Nipponbare genome assembly was updated by revising and validating the minimal tiling path of clones with the optical map for rice. Sequencing errors in the revised genome assembly were identified by re-sequencing the genome of two different Nipponbare individuals using the Illumina Genome Analyzer II/IIx platform. A total of 4,886 sequencing errors were identified in 321 Mb of the assembled genome indicating an error rate in the original IRGSP assembly of only 0.15 per 10,000 nucleotides. A small number (five) of insertions/deletions were identified using longer reads generated using the Roche 454 pyrosequencing platform. As the re-sequencing data were generated from two different individuals, we were able to identify a number of allelic differences between the original individual used in the IRGSP effort and the two individuals used in the re-sequencing effort. The revised assembly, termed Os-Nipponbare-Reference-IRGSP-1.0, is now being used in updated releases of the Rice Annotation Project and the Michigan State University Rice Genome Annotation Project, thereby providing a unified set of pseudomolecules for the rice community.

**Conclusions:**

A revised, error-corrected, and validated assembly of the Nipponbare cultivar of rice was generated using optical map data, re-sequencing data, and manual curation that will facilitate on-going and future research in rice. Detection of polymorphisms between three different Nipponbare individuals highlights that allelic differences between individuals should be considered in diversity studies.

**Electronic supplementary material:**

The online version of this article (doi:10.1186/1939-8433-6-4) contains supplementary material, which is available to authorized users.

## Background

The International Rice Genome Sequencing Project (IRGSP) completed the sequencing of the *japonica* rice cultivar Nipponbare in 2005 (International Rice Genome Sequencing Project 2005). In this project, the consortium employed a clone-by-clone sequencing strategy after construction of a minimum tiling path (MTP) for each chromosome. Subsequently, two genome assemblies were independently produced. One by the Rice Genome Annotation Project initially located at The Institute for Genomic Research and now at Michigan State University (MSU) and another by the Rice Annotation Project (RAP) (Ouyang et al. 2007; Tanaka et al. 2008). The two sets of pseudomolecules differed slightly due to differences in selection of the clones underlying the MTP and the lengths of gap insertions used by the two projects. The two genome assemblies have made it difficult for the rice community to move between the resources produced by the two annotation groups. To facilitate genomic-enabled research, a unified, single genome assembly of the Nipponbare rice reference genome was constructed by updating the MTP, validating the final MTP with optical mapping data, and error-correcting the unified assembly using next generation re-sequencing data.

The finished quality of the reference genome sequence of Nipponbare rice in 2005 was estimated to be less than one error in 10 kb (International Rice Genome Sequencing Project 2005). Recent advancements in sequencing technologies have enabled re-sequencing of multiple rice genomes. To date, several groups have performed genome-wide genetic diversity analyses among rice cultivars. The genome of an elite *japonica* rice cultivar, Koshihikari, which is closely related to Nipponbare, was sequenced using the Illumina platform and single nucleotide polymorphisms (SNPs) between Nipponbare and Koshihikari were estimated on an average to be at least one per 5.7 kb (i.e., 1.8 × 10^-4^ per site) (Yamamoto et al. 2010). Sequencing of 517 rice landraces identified approximately 3.6 million SNPs, a frequency of 9.32 per kb (Huang et al. 2010). Since the polymorphism differences between rice cultivars are limited, a high quality reference genome sequence is essential for the comparison of closely related rice cultivars (Zhang et al. 2010; Lu et al. 2010; Huang et al. 2012; Xu et al. 2012; Yang et al. 2012) so that errors can be minimized. Here, we report a single high-quality reference genome sequence for rice from the *japonica* cultivar Nipponbare (Os-Nipponbare-Reference-IRGSP-1.0) in which the MTP and bacterial artificial chromosome (BAC)/P1 artificial chromosome (PAC) assemblies were validated using optical map data. Fine-scale validation was performed using error correction and whole genome re-sequencing data obtained from two next generation platforms, the Illumina Genome Analyzer II/IIx and Roche GS FLX.

## Results and discussion

### Construction of a minimum tiling path validated by an optical map

To revise the physical map, clone orders were manually examined using an optical map of the rice genome (Zhou et al. 2007). Eight new clones (six BACs: AC151599.2, AC157835.1, AC161790.1, AC167227.1, AC157500.1, AC174464.1; one PAC: AP004805.1; one PCR product: AC150775.1) were added to the MTP. One BAC clone (AP005604.3) was found to completely overlap with other clones and was excluded. The directions of two fosmid clone sequences (AC151105.2 and AP009057.1) were resolved. Seven Syngenta contig sequences (Goff et al. 2002) were mapped to the ends of continuous sequences (contigs) or in physical gaps (Additional file 1). Fifty-three physical gaps, including novel gaps identified in this study, remained in the genome assembly (Additional file 2); note, one gap was removed by visual inspection of GS-FLX reads (see "Detection of misassembling and erroneous large gaps"). In addition, gaps in the genome sequence at 19 telomeres were annotated by the insertion of Ns at the end of the chromosomes (Additional file 2).

The final assembled genome was composed of 3,475 genomic sequences: 2,482 BACs, 901 PACs, 37 fosmids, 2 plasmids, 5 PCR products, 41 partial sequences from genomic clones, and 7 Syngenta contigs (Additional file 3). The genome size is 373,173,519 bp after error correction with Illumina whole genome re-sequencing reads (see "Detection of small sequencing errors by mapping Illumina reads"). The total gap size between contigs was estimated by fluorescence in situ hybridization (Additional file 4) to be 9,598,219 bp and 7,290,600 bp by optical mapping (Additional file 5) resulting in a total assembly size of 382,771,738 bp and 380,464,119 bp, respectively. Since the rDNA regions, which are estimated to span a total of 3.7 Mbp (Ohmido et al. 2000; Oono and Sugiura 1980), are not included in this estimate, the actual genome size of the Nipponbare cultivar is 384.2-386.5 Mbp. Thus, the new assembly covers 96.6-97.1% of the entire Nipponbare rice genome.

### Detection of small sequencing errors using Illumina-platform generated reads

To correct sequencing errors in the original reference sequence, the genomes of two different individuals of Nipponbare rice were re-sequenced at the National Institute of Agrobiological Sciences (NIAS) and Cold Spring Harbor Laboratory (CSHL). For the NIAS dataset, ~70 million 36-bp and 60 million 51-bp single-end reads, corresponding to a total of 5.6 Gbp genomic sequence, were generated (Table 1). The CSHL dataset included more than ~269 million 76-bp paired-end reads, corresponding to a total of 20.4 Gbp of sequence (Table 1). After preprocessing of these reads to filter out low quality reads, ~110 million single-end reads from NIAS, and ~247 million paired-end and ~10 million unpaired reads from CSHL remained (Table 1). To identify sequencing errors in the reference sequence as well as potential allelic sites in the NIAS and/or CSHL individuals relative to the reference sequence, all reads were mapped to the pseudomolecules using the Burrows-Wheeler Aligner (BWA) (Li and Durbin 2009). In total, ~72 million of the NIAS single-end reads and ~7 million of CSHL unpaired reads mapped uniquely to the pseudomolecules (Table 2). For the CSHL paired-end reads, ~192 million reads uniquely mapped within the estimated insert size of the paired end reads (Table 2).

**Table 1:** **Assessment and processing of re-sequencing datasets used in this study**

Library	Type^a^	Original read length (bp)	Number of reads			
			Initial purity filtered reads	Remaining after low quality trimming	Remaining after adaptor trimming	Remaining after pairing^b^
NIAS	SE	36	70,387,992	59,966,207	59,344,842	-
		51	60,164,564	51,717,577	50,981,527	-
CSHL	PE	76	269,062,790	261,197,347	257,096,019	246,779,548 (10,316,471)

^a^ SE: single-end reads, PE: paired-end reads.

^b^ The value in parenthesis is the number of unpaired reads for which one of the reads in a pair was discarded during the preprocessing to remove low quality reads.

**Table 2:** **Statistics of mapping results by BWA**

Data-set	Read type	Number of pre-processed reads	Uniquely mapped^a^	Uniquely & properly mapped^b^	Multiple^c^	Unmapped^d^
NIAS	SE, 36bp	59,344,842	38,193,181 (64.4%)	-	18,463,375 (31.1%)	302,057 (0.5%)
			[40,579,410 (68.4%)]^e^			
	SE, 51bp	50,981,527	34,124,172 (66.9%)	-	14,613,663 (28.7%)	385,876 (0.8%)
			[35,981,988 (70.6%)]			
CSHL	PE, 76bp	246,779,548	191,891,774 (77.8%)	185,630,272 (75.2%)	40,901,655 (16.6%)	13,986,119 (5.7%)
				[190,608,407 (77.2%)]		
	Unpaired PE^f^, 76bp	10,316,471	7,235,730 (70.1%)	-	1,909,048 (18.5%)	819,735 (7.9%)
			[7,587,688 (73.5%)]			

^a^ Number of uniquely mapped reads on the assembled pseudomolecules.

^b^ Number of uniquely mapped reads with proper distances between paired end reads.

^c^ Number of reads that mapped to multiple positions on the assembled pseudomolecules with the same score.

^d^ Number of reads that could not be mapped to the assembled pseudomolecules.

^e^ The numbers of reads in the square brackets includes mapped reads with lower mapping quality (MAPQ <20).

^f^ Unpaired reads for which one of the pair was discarded in the preprocessing and thus was mapped as a single-end read.

**Figure 1:**
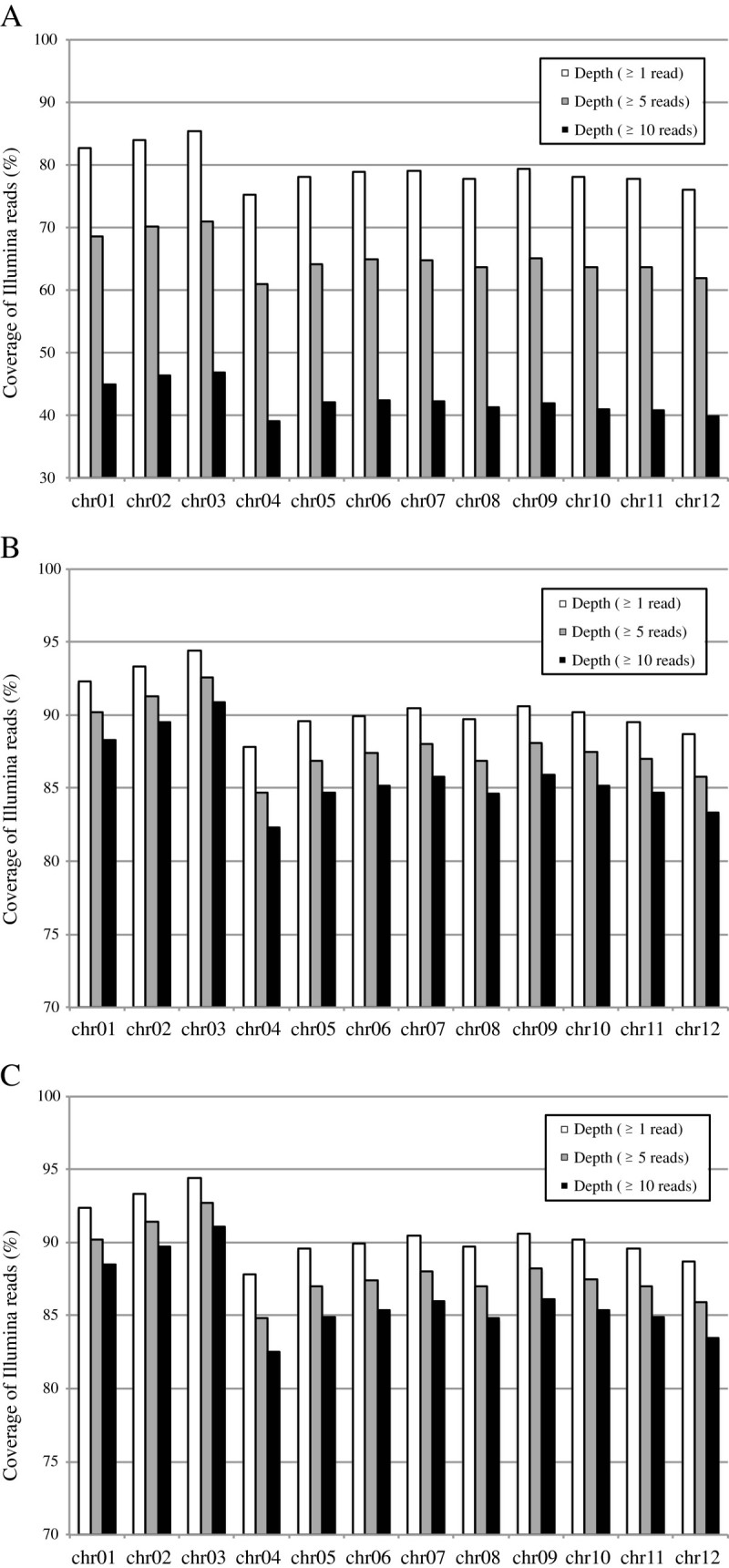
**Depth of coverage of Illumina reads on the assembled genome.** The depth of coverage of the Illumina reads at ≥1 read, ≥5 reads, and ≥10 reads are shown for the (**A**) NIAS, (**B**) CSHL, and (**C**) NIAS + CSHL datasets.

To distinguish allelic differences between the genome sequence and the NIAS and CSHL re-sequenced individuals, we examined three datasets, NIAS, CSHL, and NIAS + CSHL, that had average read depths of 7.9, 35.7, and 43.6, respectively. With respect to coverage, at least one read uniquely aligned to the pseudomolecules thereby covering 79.4% (NIAS), 90.5% (CSHL), or 90.6% (NIAS + CSHL) of the reference genome (Figure 1 and Additional file 6). Although 39% of the rice genome is occupied by repetitive sequences (Sakai and Itoh, 2010), the repetitive elements are not necessarily identical to each other, and can be distinguished by longer nucleotide stretches including flanking regions. As a result, more than 90% of the nucleotides in the pseudomolecules could be validated by at least one uniquely aligned read. Furthermore, for accurate detection of sequencing errors, we used only sites covered by 10 or more reads. The proportions of such sites were 42.4% (NIAS), 85.9% (CSHL), and 86.1% (NIAS + CSHL) (Figure 1), thereby allowing sequencing errors to be assessed at more than 86% of the genomic sites with high coverage (Table 3). As the original genome assembly size was ~373 Mbp, a total of ~321 Mbp could be evaluated for sequencing errors.

**Table 3:** **Coverage and number of effective sites for detection of variation**

Chr	length (bp)^a^	NIAS		CSHL		NIAS + CSHL	
		Cov^b^ (%)	Eff^c^ (%)	Cov (%)	Eff (%)	Cov (%)	Eff (%)
chr01	43,270,899	35,791,098 (82.7%)	18,246,140 (42.2%)	39,956,568 (92.3%)	38,068,615 (88.0%)	39,961,540 (92.4%)	38,175,168 (88.2%)
chr02	35,937,247	30,154,356 (83.9%)	15,638,850 (43.5%)	33,524,321 (93.3%)	32,062,712 (89.2%)	33,528,628 (93.3%)	32,148,332 (89.5%)
chr03	36,413,819	31,059,089 (85.3%)	15,977,692 (43.9%)	34,365,834 (94.4%)	32,996,819 (90.6%)	34,369,143 (94.4%)	33,082,516 (90.9%)
chr04	35,502,790	26,736,290 (75.3%)	12,921,248 (36.4%)	31,176,439 (87.8%)	29,064,227 (81.9%)	31,186,864 (87.8%)	29,153,050 (82.1%)
chr05	29,958,438	23,400,797 (78.1%)	11,810,143 (39.4%)	26,853,884 (89.6%)	25,256,988 (84.3%)	26,857,426 (89.6%)	25,327,281 (84.5%)
chr06	31,248,789	24,651,481 (78.9%)	12,398,636 (39.7%)	28,084,165 (89.9%)	26,516,110 (84.9%)	28,088,052 (89.9%)	26,594,708 (85.1%)
chr07	29,697,629	23,447,981 (79.0%)	11,725,346 (39.5%)	26,880,079 (90.5%)	25,363,522 (85.4%)	26,883,654 (90.5%)	25,438,043 (85.7%)
chr08	28443,027	22,122,986 (77.8%)	11,013,746 (38.7%)	25,508,624 (89.7%)	23,953,312 (84.2%)	25,512,422 (89.7%)	24,024,380 (84.5%)
chr09	23,012,721	18,240,332 (79.3%)	9,052,990 (39.3%)	20,858,168 (90.6%)	19,681,323 (85.5%)	20,860,908 (90.6%)	19,739,054 (85.8%)
chr10	23,208,246	18,119,906 (78.1%)	8,873,977 (38.2%)	20,931,379 (90.2%)	19,673,976 (84.8%)	20,934,165 (90.2%)	19,732,853 (85.0%)
chr11	29,021,139	22,586,659 (77.8%)	11,082,625 (38.2%)	25,987,560 (89.5%)	24,468,775 (84.3%)	25,990922 (89.6%)	24,546,522 (84.6%)
chr12	27,531,905	20,923,037 (76.0%)	10,270,579 (37.3%)	24,427,691 (88.7%)	22,818,854 (82.9%)	24,432,616 (88.7%)	22,891,243 (83.1%)
Total	373,246,649	297,234,012 (79.6%)	149,011,972 (39.9%)	338,554,712 (90.7%)	31,9925,233 (85.7%)	338,606,340 (90.7%)	320,853,150 (86.0%)

^a^ Length before error correction.

^b^ Cov: Number of sites covered with at least one read.

^c^ Eff: Number of effective sites covered with at least 10 high quality (base quality ≥20) reads.

We classified all nucleotides into five categories: "reference type," "sequencing error," "allele (within an individual)," "allelic difference between individuals," and "low depth" (see Methods and Additional file 7). The classification is based on the type and frequency of nucleotide at each site for the three datasets. Our survey of SNP-type sequencing errors detected 3,447 sites that showed high frequencies of non-reference type bases and were classified into "sequencing error" (Additional file 8 and Additional file 9). Since BWA generates gapped alignments, small insertion/deletion (indel) type variants (1–4 bp gaps) could also be detected: 642 sites were insertions and 797 were deletions. Thus, 4,886 errors were found in the 321 Mbp of the reassembled reference genome. The IRGSP estimated that the error rate of the reference genome sequence was less than one per 10,000 nucleotides (International Rice Genome Sequencing Project 2005). In fact, the average frequency of sequencing errors estimated by our Illumina re-sequencing data was 0.15 errors per 10,000 nucleotides, substantially lower than the estimation by the IRGSP. While this may be an under-estimation of the error rate due to our inability to map reads to all nucleotides in the assembled rice genome and the lack of the requisite read depth at every nucleotide, we were able to assess 321 of the 373 Mb assembly for errors.

**Figure 2:**
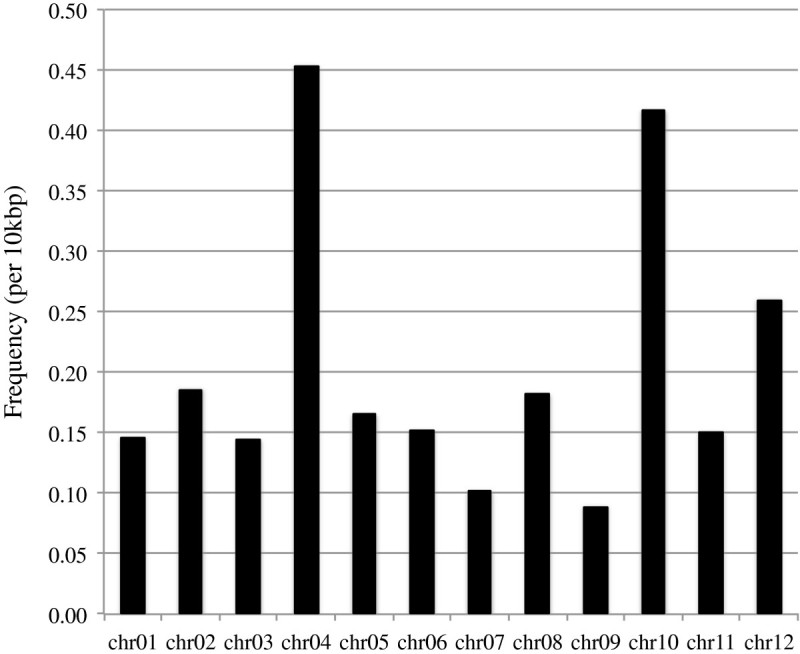
**Frequency of allelic sites per 10 kb among the 12 rice chromosomes.**

The re-sequencing analysis by Illumina reads also revealed that a number (6,433) of the sites had allelic differences in the datasets. The frequency of the allelic sites was on average 0.20 in 10,000 nucleotides (Figure 2), which may not be negligible for genome sequence comparison between cultivars. Chromosomes 4 and 10 are the most repetitive chromosomes in the rice genome (International Rice Genome Sequencing Project 2005) and have larger sequencing error rates than other chromosomes (Figure 2). Thus, the higher allele frequency detected for these two chromosomes may be attributable in part to sequencing error. In this analysis, the same rice cultivar (Nipponbare) was used in the NIAS and CSHL re-sequencing as was used in the original IRGSP sequencing effort. However, the Nipponbare rice NIAS and CSHL individuals were derived from different Nipponbare populations preserved in different laboratories and have been propagated independently for an unknown number of generations. Thus, these two individuals may have accumulated mutations after multiple generations as described previously in *Arabidopsis thaliana* (Ossowski et al. 2010). Comparison between the CSHL and NIAS datasets indicated that 250 sites differed between the NIAS and CSHL individuals and were not allelic to each other (Additional file 10). Hence, the occurrence frequency of the allelic sites is 0.0078 per 10,000 nucleotides. It should also be noted that some of these differences may be derived from somatic mutations as the DNA used for sequencing was derived from young leaves two weeks after germination. Residual heterozygosity in recombinant inbred lines has been reported in maize (McMullen et al. 2009; Eichten et al. 2011) and as the IRGSP employed a clone-by-clone approach, only a single clone (and homolog) was selected for sequencing. Thus, if residual heterozygosity were present in the original individual used in the IRGSP sequencing effort, only a single allele would be represented in the reference assembly. Indeed, we were able to identify a small subset of allelic sites within individuals (Additional file 11). Therefore, caution regarding the origin of allelic differences should be considered when conducting re-sequencing studies. Allelic SNPs within an individual (5,412) and between individuals (232) were classified into six groups (Nonsense, Missense, Silent, UTR, Intron and Intergenic) based on the RAP-DB gene models. The majority (79.4%) of allelic sites were located in intergenic regions (Additional file 12) with only 3.6% of allelic variations with the potential for affecting the encoded amino acid.

### Detection of misassembly

Long read sequences were generated using the Roche GS FLX platform and used to identify misassembly of BAC/PAC clones as well as large indel sequencing errors. A total of 1.0 Gbp from 2,706,353 reads was produced. The reads were filtered to remove low quality reads, yielding 2,705,634 reads with an average read length of 377 bp. To detect large gaps in the reference assembly, these reads were aligned to the pseudomolecules using Megablast (Zhang et al. 2000). We selected reads that mapped unambiguously to two different chromosomal positions within a 1 Mbp interval and where the total of two high scoring pairs reported by Megablast covered >90% of the original read. From this initial set of alignments, 205 reads were found to meet these criteria. A total of 200 of these reads were removed through manual inspection and five large indel gaps were retained. Three were insertions, indicating that additional nucleotides should be inserted into the reference genome, while the other two were deletions. The two deletion errors were further validated by mapping the Illumina reads to the long reads that matched these regions. In one case, a physical gap that had originally been inserted into the reference was found to be unnecessary based on our long read validation, and this gap was removed. For the other four cases, the erroneous indels were located near tandem repetitive sequences.

### Nomenclature, data availability and annotation of the Os-Nipponbare-Reference-IRGSP-1.0 genome

We have named this updated assembly "Os-Nipponbare-Reference-IRGSP-1.0" to signify that it is from rice (*Oryza sativa*), the Nipponbare cultivar, a high quality reference assembly, from the IRGSP, and version 1.0. We envision that future assemblies of rice will be of draft quality and from other entities, and as is the case with the Nipponbare rice genome, will be updated as new sequencing datasets become available in the future. We are proposing this nomenclature for other rice genome assemblies as an informative way for the community to readily interpret the origin, quality, and iteration of rice genome sequences. One objective in this study was to provide a single unified set of pseudomolecules for two parallel annotation efforts, the RAP (Tanaka et al. 2008, http://rapdb.dna.affrc.go.jp/) and the MSU Rice Genome Annotation Project (Ouyang et al. 2007, http://rice.plantbiology.msu.edu/) in which both annotation projects have now updated their annotation with the underlying Os-Nipponbare-Reference-IRGSP-1.0 pseudomolecules and provided this via their project websites.

The annotation of the Rice Annotation Project Database (RAP-DB) was updated for Os-Nipponbare-Reference-IRGSP-1.0. A total of 4,993 loci from the RAP annotation on the IRGSP build 5 genome were deprecated in this release. Of those, 4,766 (95%) loci became obsolete due to the overlaps with repetitive sequences on the new pseudomolecules. The exon-intron structures of genes and their splicing variants were identified or predicted as described previously (Tanaka et al. 2008). As a result, 37,872 loci, of which 35,681 have protein-coding potential, were determined. In addition, 6,642 variants of alternative splicing were found. Furthermore, a number of functional annotations were carefully examined through literature surveys. Manual curation efforts improved the gene structures and functional descriptions of 97 loci. All the information including the repeat-masked genome assembly can be downloaded from the RAP-DB (http://rapdb.dna.affrc.go.jp/download/irgsp1.html). For users' convenience, previous versions based on obsolete genome assemblies are also available at the RAP-DB Legacy database (http://rapdblegacy.dna.affrc.go.jp/).

Annotated genes and gene models from the MSU Rice Genome Annotation Project (Ouyang et al. 2007) were transferred from the previous MSU pseudomolecule build to the Os-Nipponbare-Reference-IRGSP-1.0 pseudomolecules and functional annotation updated to reflect new evidence datasets. The current release of the MSU Rice Genome Annotation Project (Release 7) based on the Os-Nipponbare-Reference-IRGSP-1.0 pseudomolecules contains 56,081 loci encoding 66,433 gene models. Of these, 39,102 loci (49,110 gene models) are non-transposable element related. A total of 1,240 loci from the MSU Release 6.1 annotation set were deprecated in this new release. Functional annotation of the loci involved searches against the UniRef 100 database, Pfam domain database, Interpro and alignments to a wide range of expression profile datasets including Sanger-derived Expressed Sequence Tags, full-length cDNAs, Massively Parallel Signature Sequences, Digital Gene Expression, and mRNA-seq datasets (http://rice.plantbiology.msu.edu/expression.shtml). The Rice Genome Annotation Project Release 7 database and tools are available for searching, download, and analysis at http://rice.plantbiology.msu.edu/. An install of the Generic Genome Browser (Stein et al. 2002) providing 83 tracks of annotation including the MSU Rice Genome Annotation Project Release 7 data is available at http://rice.plantbiology.msu.edu/cgi-bin/gbrowse/rice/.

Comparison of the RAP and MSU annotation datasets show a high degree of concordance between the two annotations with 33,708 loci overlapping by at least 1bp between the two annotation sets. However, as the two approaches weight *ab initio* gene predictions and transcript/protein evidence differently (Ouyang et al. 2007, Tanaka et al. 2008), there are differences in the two datasets with more protein-coding genes predicted in the MSU than the RAP annotation.

## Conclusions

The genome assembly of *Oryza sativa* (cv. Nipponbare), which had been constructed and provided independently by two groups, was unified. Sequencing errors were thoroughly examined so that in-depth analyses among rice cultivars will be possible. Furthermore, our survey of alleles found variations within the Nipponbare cultivar that are mostly attributable to outcrossing, residual heterozygosity, and/or somatic mutations through the standard process of propagation in individual laboratories. Our ability to detect a number of allelic differences in the three Nipponbare rice individuals surveyed suggests that allelic differences should not be readily dismissed in SNP assessments of rice diversity. The high-quality reference genome assembly presented here is an invaluable resource for studies of emerging re-sequencing data and is available in the RAP-DB (http://rapdb.dna.affrc.go.jp/) and the MSU Rice Genome Annotation Project (http://rice.plantbiology.msu.edu/) websites.

## Methods

### Sequence data sources

The IRGSP clone and PCR sequences of the *O*. *sativa* (*japonica* group, cultivar Nipponbare) genome deposited in the International Nucleotide Sequence Databases as of 25 February 2010 were used in construction of the MTP. In addition, sequence reads generated by the Syngenta rice genome sequencing project (Goff et al. 2002) were assembled and used to extend contigs.

For the next-generation DNA sequencing of an NIAS individual, total genomic DNA was prepared from nuclei isolated from Nipponbare rice young leaves (two weeks after germination) using the CTAB method (Murray and Thompson 1980). The DNA samples were fragmented by a nebulizer or Branson Sonifier 250 (Danbury, CT). Sequencing libraries were constructed following the protocols with Illumina Genomic DNA Sample Preparation Kit and Roche GS DNA Library Preparation Kit, respectively. Illumina genome sequencing was performed by Illumina Genome Analyzer II/IIx with the Illumina version 2 sequencing kit. GS-FLX genome sequencing was performed using the Roche GS LR70 Sequencing Kit. The sequence reads are available at the DDBJ Sequence Read Archive (DRA000651).

For the CSHL individual, ~5 μg of Nipponbare rice genomic DNA was used as input for standard Illumina libraries. The DNA was sheared by adaptive focused acoustics using the Covaris (Woburn, MA) instrument and end-repaired using T4 DNA polymerase, Klenow fragment, and T4 polynucleotide kinase. Fragments were then treated with Klenow fragment (3’ - 5’ exonuclease) to add a single 3’ deoxyA overhang and ligated to standard paired-end Illumina adapters. Qiagen (Valencia, CA) columns were used for purification between steps. The fragments were size-selected at ~225 bp (including adapters) using agarose gel electrophoresis. The actual insert size excluding adapters was ~150 bp. The library was then PCR amplified using Phusion DNA polymerase in HF buffer for 14 cycles and quantified using the Agilent BioAnalyzer (Santa Clara, CA). All libraries were normalized to 10 nM before loading on the Illumina sequencers. Production sequencing was performed using Illumina GAIIx instruments with paired-end modules using the Illumina version 3 sequencing kits. The library was sequenced with 76 bp paired-end read lengths. Sequence data was processed using the Illumina GAPipeline v1.1 and v1.3.2 (Firecrest/Bustard v1.9.6 and Firecrest/Bustard v1.3.2). The sequence reads are available at the Sequence Read Archive of NCBI (SRX032913).

Syngenta rice genome sequences (Goff et al. 2002) were filtered by using IRGSP rice genomic sequences with similarity searches. The filtered sequences were then assembled; 50 large Syngenta contigs (between 4 kb and 40 kb), a total of 748 kb were used for potential gap filling.

### Construction of the genome assembly based on a minimum tiling path and validation with the optical map

The MTP of the IRGSP clones for each of the 12 pseudomolecules were updated prior to validation with the optical map. We manually checked information on the clone order and clone overlaps in the IRGSP physical map. For the overlapping regions, phase 3 BAC/PAC sequences, which have finished, quality sequences without gaps (see http://www.ncbi.nlm.nih.gov/projects/genome/glossary.shtml), were preferentially chosen. Any ambiguous nucleotides, such as Rs and Ys, were converted to Ns. The lengths of physical gap between contigs were estimated by FISH (International Rice Genome Sequencing Project 2005). A total of 1,000 Ns were inserted in at each physical gap. Other gaps in the original entries were left unchanged.

Sequences of 4,005 rice BACs/PACs, 50 Syngenta contigs, and the 12 pseudomolecules based on the aforementioned MTP were digested *in silico* by SwaI. The sequences were then aligned (Valouev et al. 2006) against the Nipponbare rice optical map (Zhou et al. 2007) to automatically reveal any map-sequence discordances. The alignments were graphically viewed using GnomSpace (Teague et al., 2010). The alignments of all 12 pseudomolecules, along with the BACs/PACs, were manually examined. In general, the pseudomolecules were in good concordance with the optical map. There were 169 discordances between the optical map and the pseudomolecules, including missing SwaI sites, extra SwaI sites, missing sequence, extra sequence, complex events, and potential misassembly. When there was a discordance, a modification (changing BAC/PAC/Syngenta contig, or adding/eliminating a gap) was made on the MTP when:

1. There was another BAC/PAC sequence that aligned better against the optical map;
2. A BAC/PAC/Syngenta contig extended the sequence into a physical gap;
3. A reverse orientation of a BAC/PAC was aligned better against the optical map;
4. A BAC/PAC/Syngenta contig was aligned perfectly against the optical map within a physical gap of a pseudomolecule;
5. A new gap was clearly identified between two neighboring clones;
6. A gap was eliminated when an overlap of two clones that were supposed to flank a gap was found.

In total, there were 23 modifications made in the tiling paths of the 12 chromosomes. Additional discordances were derived from the sequences of the BACs/PACs, rather than from the pseudomolecule construction; no attempt was made to correct the assembly of the BAC/PAC sequences to match the optical maps.

The lengths of rDNA regions were estimated to span 0.2 Mbp for 5S rDNA on chromosome 11 (Ohmido et al. 2000) and 3.5 Mbp for 17S and 25S rDNA on chromosome 9 (620 daltons/bp; Oono and Sugiura 1980) in a haploid set.

### Error corrections with Illumina and 454 reads

To detect small sequencing errors (single nucleotides and indels of 1–4 bases) in the newly assembled reference genome, Illumina Genome Analyzer II/IIx generated reads were used. The genomes of two different individuals of Nipponbare were independently re-sequenced at NIAS and CSHL. Low quality bases (<Q20) were trimmed from both 5’- and 3’ ends of the read until two or more consecutive bases with a high quality score (≥Q20) were observed. Next, the Illumina adapter sequences (5' P-GATCGGAAGAGCGGTTCAGCAGGAATGCCGAG) and (5’ ACACTCTTTCCCTACACGACGCTCTTCCGATCT) were removed using the fastx_clipper, which is part of the FASTX-Toolkit (http://hannonlab.cshl.edu/fastx_toolkit/). Reads that were <32 bp in length were discarded for further analyses. If only one read of a paired-end read set was discarded in these preprocessing steps, the other read was regarded as a single-end read and named "unpaired." All qualified reads were aligned to the reference genome using BWA v0.5.8a with default options (Li and Durbin 2009). The NIAS single-end reads and CSHL unpaired reads were aligned in the single-end mode using the BWA command “samse”. The CSHL paired-end reads were aligned in the paired-end mode using the BWA command “sampe”. The reads that matched to multiple genomic positions were discarded. A pile up alignment file of all uniquely mapped reads with a mapping quality value of ≥20 was generated using SAMtools v1.8 (Li et al. 2009). To avoid erroneous detection of variants, only sites with a read depth of 10 or more were selected.

By comparing the Illumina reads with the reference genome, each aligned site was first classified into four categories: "reference type (R)," "non-reference type (N)," "allelic (A)," and "low depth (L)" for each of three sets (NIAS, CSHL and NIAS + CSHL) (Additional file 7). If a site had less than 10 reads, the site was "low depth (L)," which means we were unable to assess the site due to low sampling. If ≥80% of the reads were identical to the reference base, the site was classified as "reference type (R)". If ≥80% of the reads were discordant with the reference base, the site was classified as "non-reference type (N)". If there were two alleles with ≥40% read support, the site was classified as "allelic (A)". Since we have two data sets from NIAS and CSHL, the classifications of the three sets (NIAS, CSHL and NIAS + CSHL) were combined and reexamined to decide the genotype for each site (Additional file 7): "reference type", "sequencing error (Additional file 9)", "alleles between individuals” (Additional file 10), "alleles within individuals” (Additional file 11), and "low depth". SNPs classified as allelic variations were annotated based on the RAP-DB gene models using SnpEff v. 3.1 (Cingolani et al. 2012) (Additional file 12).

The genome of the same NIAS individual used in the Illumina re-sequencing was sequenced using the Roche GS FLX platform. Low quality bases (<Q20) were trimmed by the same method as that for the Illumina reads. Repetitive sequences were detected in each read using RepeatMasker Open-3.0 (http://www.repeatmasker.org/) with the MIPS Repeat Element Database (mips-REdat) version 4.3 (http://mips.helmholtz-muenchen.de/plant/genomes.jsp; Spannagl et al. 2007) and the Triticeae Repeat Sequence Database release 10 (http://wheat.pw.usda.gov/ITMI/Repeats/). All preprocessed reads were aligned to the reference genome using Megablast (version 2.2.24) with the following options: -F 'm D' -U T -e 1e-10 (Zhang et al. 2000).

### Re-validation and annotation of final assembly

The final, error-corrected pseudomolecules were virtually digested with SwaI and aligned against the rice optical map. A total of 144 major discordances were annotated in the current build of rice pseudomolecules (Additional file 5). Among these discordances, there were 53 physical gaps, including 19 telomeric gaps. The sizes of the gaps range from 0.6 kb to 2.4 Mb, measured by adding up the sizes of the un-matched optical fragments at the gap locations. The discordances were grouped into 5 classes:

Class 1: Physical gaps in the pseudomolecule;

Class 2: Missed fragment or extra fragment: only the discordances with ≥5 kb missed/extra fragment(s) were annotated;

Class 3: Significant size difference: mostly the size differences were ≥5 kb;

Class 4: Multiple different SwaI sites in the same area;

Class 5: Multiple different SwaI sites and multiple un-matched SwaI fragments. The total sizes of the pseudomolecule and the optical map at the location were not comparable, indicating possible misassembly).

## Authors’ information

**Nipponbare Genome Re**-**sequencing Project**

***Genome sequencing and assembly team***

Yoshihiro Kawahara, Melissa de la Bastide, John P Hamilton, Hiroyuki Kanamori, W Richard McCombie, Shu Ouyang, David C Schwartz, Tsuyoshi Tanaka, Jianzhong Wu and Shiguo Zhou.

***MSU annotation team***

Kevin L Childs, Rebecca M Davidson, Haining Lin, Lina Quesada-Ocampo and Brieanne Vaillancourt.

***RAP annotation team***

Hiroaki Sakai, Sung Shin Lee, Jungsok Kim and Hisataka Numa.

***Coordinators***

Takeshi Itoh, C Robin Buell and Takashi Matsumoto.

## Electronic supplementary material

Additional file 1:**Table S1.** Newly inserted Syngenta sequences. (DOC 30 KB)

Additional file 2:**Table S2.** Statistics of the unified reference genome. (DOC 42 KB)

Additional file 3:**Table S3.** Sources of clones in the minimum tiling path. (DOC 42 KB)

Additional file 4:**Table S4.** Positions and sizes of gaps in the unified reference genome. (DOC 76 KB)

Additional file 5:**Table S5.** Major discordances annotated in the current build of rice pseudomolecules. (XLS 47 KB)

Additional file 6:**Figure S1.** Depth of coverage of Illumina reads on the assembled genome. (TIFF 740 KB)

Additional file 7:**Figure S2.** Classification of each site in the error corrections/SNP detections by Illumina reads. (TIFF 1 MB)

Additional file 8:**Table S6.** Number of sequencing errors, alleles between and within individuals for different thresholds of read depth. (XLS 36 KB)

Additional file 9:**Figure S3.** An example case of “sequencing error”. (TIFF 3 MB)

Additional file 10:**Figure S4.** An example case of “alleles between individuals (NIAS and CSHL). (TIFF 3 MB)

Additional file 11:**Figure S5.** An example case of “alleles within individuals”. (TIFF 3 MB)

Additional file 12:**Table S7.** Classification of SNP-type allelic variations. (XLS 32 KB)

Below are the links to the authors’ original submitted files for images.

Authors’ original file for figure 1

Authors’ original file for figure 2

## Abbreviations

BAC: Bacterial Artificial Chromosome
BWA: Burrows-Wheeler Aligner
CSHL: Cold Spring Harbor Laboratory
DDBJ: DNA Data Bank of Japan
IRGSP: International Rice Genome Sequencing Project
MSU: Michigan State University
MTP: Minimum Tiling Path
NIAS: National Institute of Agrobiological Sciences
NCBI: National Center for Biotechnology Information
PAC: P1 Artificial Chromosome
RAP: Rice Annotation Project
SNP: Single Nucleotide Polymorphism.
