# Supplementary material for: Improvement of the Oryza sativa Nipponbare reference genome using next generation sequence and optical map data
Source: Rice (N Y). 2013 Feb 6;6:4. doi: 10.1186/1939-8433-6-4 (PMC5395016; doi:10.1186/1939-8433-6-4)
Supplement: Supplementary file 1 — Additional file 1:Table S1. Newly inserted Syngenta sequences. (DOC 30 KB) [file 12284_2012_41_MOESM1_ESM.doc]

Table S1. Newly inserted Syngenta sequences

| **Sequence ID** | **Position** | **Note** |
| --- | --- | --- |
| Syng_TIGR_001 | chr01:11495818-11510114:+ | **-** |
| Syng_TIGR_003 | chr03:16363799-16386795:+ | This sequence was inserted in a physical gap and split it into two gaps |
| Syng_TIGR_018 | chr03:31113057-31123515:+ | This sequence was inserted in a physical gap and split it into two gaps |
| Syng_TIGR_017 | chr10:4226557-4243136:+ | **-** |
| Syng_TIGR_040 | chr10:7745102-7754835:+ | **-** |
| Syng_TIGR_025 | chr11:8925804-8942193:+ | **-** |
| Syng_TIGR_006 | chr11:13390687-13410588:- | **-** |
