# Supplementary material for: Improvement of the Oryza sativa Nipponbare reference genome using next generation sequence and optical map data
Source: Rice (N Y). 2013 Feb 6;6:4. doi: 10.1186/1939-8433-6-4 (PMC5395016; doi:10.1186/1939-8433-6-4)
Supplement: Supplementary file 2 — Additional file 2:Table S2. Statistics of the unified reference genome. (DOC 42 KB) [file 12284_2012_41_MOESM2_ESM.doc]

**Table S2. Statistics of the unified reference genome**

|  | Contig length (bp) | Estimated gap length (bp) | Estimated Chromosome length (bp) | Physical gaps | Telomere gap | GC (%) |
| --- | --- | --- | --- | --- | --- | --- |
| chr01 | 43,262,923 | 1,775,705 | 45,038,628 | 6 | 2 | 43.8 |
| chr02 | 35,932,250 | 860,000 | 36,792,250 | 4 | 1 | 43.3 |
| chr03 | 36,405,819 | 906,548 | 37,312,367 | 7 | 1 | 43.7 |
| chr04 | 35,493,694 | 430,000 | 35,923,694 | 8 | 1 | 44.2 |
| chr05 | 29,953,434 | 120,000 | 30,073,434 | 4 | 1 | 43.9 |
| chr06 | 31,244,787 | 880,000 | 32,124,787 | 2 | 2 | 43.6 |
| chr07 | 29,694,621 | 630,000 | 30,324,621 | 2 | 1 | 43.5 |
| chr08 | 28,440,022 | 90,000 | 28,530,022 | 1 | 2 | 43.4 |
| chr09 | 23,005,720 | 890,000 | 23,895,720 | 5 | 2 | 43.5 |
| chr10 | 23,198,287 | 682,262 | 23,880,549 | 7 | 2 | 43.6 |
| chr11 | 29,015,106 | 2,183,704 | 31,198,810 | 4 | 2 | 42.9 |
| chr12 | 27,526,856 | 150,000 | 27,676,856 | 3 | 2 | 43.0 |
| Total | 373,173,519 | 9,598,219 | 382,771,738 | 53 | 19 | 43.6 |
