# Supplementary material for: Improvement of the Oryza sativa Nipponbare reference genome using next generation sequence and optical map data
Source: Rice (N Y). 2013 Feb 6;6:4. doi: 10.1186/1939-8433-6-4 (PMC5395016; doi:10.1186/1939-8433-6-4)
Supplement: Supplementary file 3 — Additional file 3:Table S3. Sources of clones in the minimum tiling path. (DOC 42 KB) [file 12284_2012_41_MOESM3_ESM.doc]

**Table S3. Sources of clones in the minimum tiling path.**

|  | BAC^a^ | PAC^b^ | Fosmid^c^ | Plasmid | PCR | Genomic fragment^d^ | Syngenta fragment | Total |
| --- | --- | --- | --- | --- | --- | --- | --- | --- |
| chr01 | 138 | 248 | 6 | 0 | 0 | 0 | 1 | 393 |
| chr02 | 236 | 116 | 5 | 1 | 0 | 1 | 0 | 359 |
| chr03 | 315 | 1 | 3 | 0 | 0 | 10 | 2 | 331 |
| chr04 | 292 | 2 | 2 | 0 | 0 | 0 | 0 | 296 |
| chr05 | 213 | 66 | 7 | 0 | 0 | 0 | 0 | 286 |
| chr06 | 111 | 167 | 1 | 0 | 0 | 2 | 0 | 281 |
| chr07 | 183 | 102 | 2 | 0 | 0 | 2 | 0 | 289 |
| chr08 | 162 | 113 | 3 | 0 | 0 | 0 | 0 | 278 |
| chr09 | 145 | 71 | 5 | 0 | 0 | 2 | 0 | 223 |
| chr10 | 182 | 1 | 1 | 1 | 0 | 21 | 2 | 208 |
| chr11 | 240 | 12 | 2 | 0 | 4 | 2 | 2 | 262 |
| chr12 | 265 | 2 | 0 | 0 | 1 | 1 | 0 | 269 |
| Total | 2,482 | 901 | 37 | 2 | 5 | 41 | 7 | 3,475 |

^a^ BACs were provided by Clemson University Genomics Institute, Rice Genome Research Program of Japan and Monsanto.

^b^ PACs were provided by Rice Genome Research Program of Japan.

^c^ Fosmids were provided by Arizona Genomics Institute.

^d^ Partial sequences of genomic clones.
