# Supplementary material for: Improvement of the Oryza sativa Nipponbare reference genome using next generation sequence and optical map data
Source: Rice (N Y). 2013 Feb 6;6:4. doi: 10.1186/1939-8433-6-4 (PMC5395016; doi:10.1186/1939-8433-6-4)
Supplement: Supplementary file 4 — Additional file 4:Table S4. Positions and sizes of gaps in the unified reference genome. (DOC 76 KB) [file 12284_2012_41_MOESM4_ESM.doc]

**Table S4. Positions and sizes of gaps in the unified reference genome**

| **Position** | **Estimated gap length by FISH** | **Type** |
| --- | --- | --- |
| chr01:1-1000 | - | Telomere |
| chr01:10053456-10054455 | 50,000 | Physical |
| chr01:11494818-11495817 | 95,705 | Physical |
| chr01:14990775-14991774 | 90,000 | Physical |
| chr01:16847195-16848194 | 1,400,000 | Physical (Centromere) |
| chr01:25477690-25478689 | 100,000 | Physical |
| chr01:40297678-40298677 | 40,000 | Physical |
| chr01:43269924-43270923 | - | Telomere |
| chr02:5969519-5970518 | 30,000 | Physical |
| chr02:13681864-13682863 | 720,000 | Physical (Centromere) |
| chr02:18795366-18796365 | 80,000 | Physical |
| chr02:24815509-24816508 | 30,000 | Physical |
| chr02:35936251-35937250 | - | Telomere |
| chr03:1-1000 | - | Telomere |
| chr03:4867172-4868171 | 50,000 | Physical |
| chr03:13464661-13465660 | 500,000 | Physical |
| chr03:16362799-16363798 | 87,005 (split by Syng_TIGR_003) | Physical |
| chr03:16386796-16387795 |  | Physical |
| chr03:19544628-19545627 | 180,000 | Physical (Centromere) |
| chr03:31112057-31113056 | 89,543 (split by Syng_TIGR_018) | Physical |
| chr03:31123516-31124515 |  | Physical |
| chr04:1-1000 | - | Telomere |
| chr04:932184-933183 | unknown | Physical |
| chr04:8427658-8428657 | 60,000 | Physical |
| chr04:9348993-9349992 | unknown | Physical |
| chr04:15655595-15656594 | unknown | Physical |
| chr04:22046964-22047963 | 300,000 | Physical |
| chr04:22098844-22099843 | unknown | Physical |
| chr04:22273447-22274446 | 10,000 | Physical |
| chr04:35463349-35464348 | 60,000 | Physical |
| chr05:7361109-7362108 | 30,000 | Physical |
| chr05:17141990-17142989 | 30,000 | Physical |
| chr05:21551064-21552063 | 40,000 | Physical |
| chr05:27174270-27175269 | 20,000 | Physical |
| chr05:29957435-29958434 | - | Telomere |
| chr06:1-1000 | - | Telomere |
| chr06:10579630-10580629 | 70,000 | Physical |
| chr06:15423583-15424582 | 810,000 | Physical (Centromere) |
| chr06:31247788-31248787 | - | Telomere |
| chr07:1-1000 | - | Telomere |
| chr07:11609585-11610584 | 330,000 | Physical |
| chr07:12185864-12186863 | 300,000 | Physical (Centromere) |
| chr08:1-1000 | - | Telomere |
| chr08:11171225-11172224 | 90,000 | Physical |
| chr08:28442023-28443022 | - | Telomere |
| chr09:1-1000 | - | Telomere |
| chr09:2787792-2788791 | 620,000 | Physical (Centromere) |
| chr09:11419064-11420063 | 40,000 | Physical |
| chr09:13605666-13606665 | 50,000 | Physical |
| chr09:20279471-20280470 | 90,000 | Physical |
| chr09:20406894-20407893 | 90,000 | Physical |
| chr09:23011721-23012720 | - | Telomere |
| chr10:1-1000 | - | Telomere |
| chr10:4094914-4095913 | unknown | Physical |
| chr10:4225557-4226556 | unknown | Physical |
| chr10:7174025-7175024 | 50,000 | Physical |
| chr10:7744102-7745101 | 50,265 | Physical |
| chr10:8169469-8170468 | 420,000 | Physical (Centromere) |
| chr10:9880808-9881807 | 61,997 | Physical |
| chr10:12197537-12198536 | 100,000 | Physical |
| chr10:23206288-23207287 | - | Telomere |
| chr11:1-1000 | - | Telomere |
| chr11:7313360-7314359 | 20,000 | Physical |
| chr11:8924804-8925803 | 43,607 | Physical |
| chr11:12179030-12180029 | 2,090,000 | Physical (Centromere) |
| chr11:13410589-13411588 | 30,097 | Physical |
| chr11:29020107-29021106 | - | Telomere |
| chr12:1-1000 | - | Telomere |
| chr12:12010056-12011055 | 150,000 | Physical (Centromere) |
| chr12:16625409-16626408 | unknown | Physical |
| chr12:20552453-20553452 | unknown | Physical |
| chr12:27530857-27531856 | - | Telomere |
| **Total estimated gap length** | **9,598,219** |  |
