# Supplementary figures and images for: Improvement of the Oryza sativa Nipponbare reference genome using next generation sequence and optical map data
Source: Rice (N Y). 2013 Feb 6;6:4. doi: 10.1186/1939-8433-6-4 (PMC5395016; doi:10.1186/1939-8433-6-4)

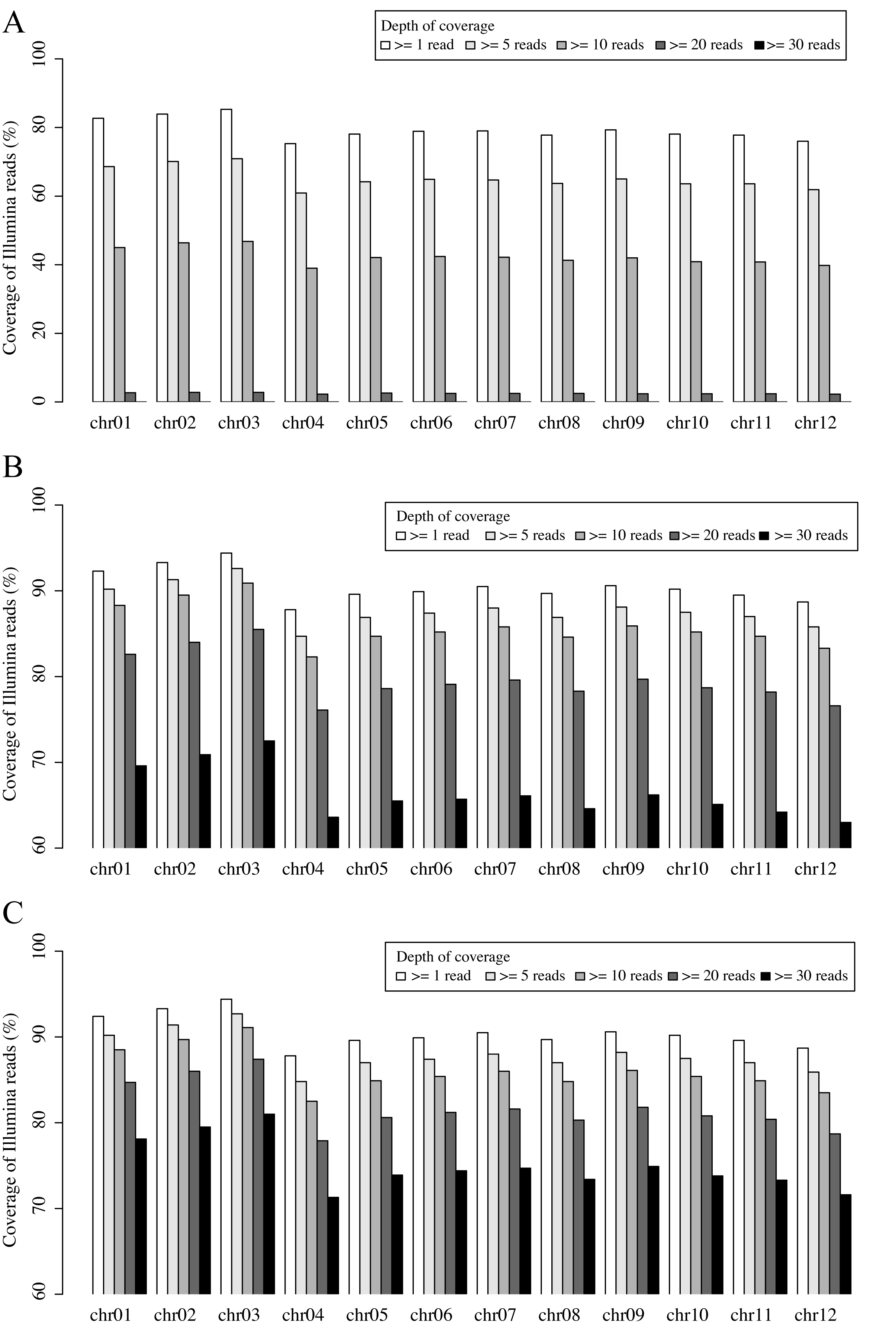

Supplement: Supplementary file 6 — Additional file 6:Figure S1. Depth of coverage of Illumina reads on the assembled genome. (TIFF 740 KB) [file 12284_2012_41_MOESM6_ESM.tiff]

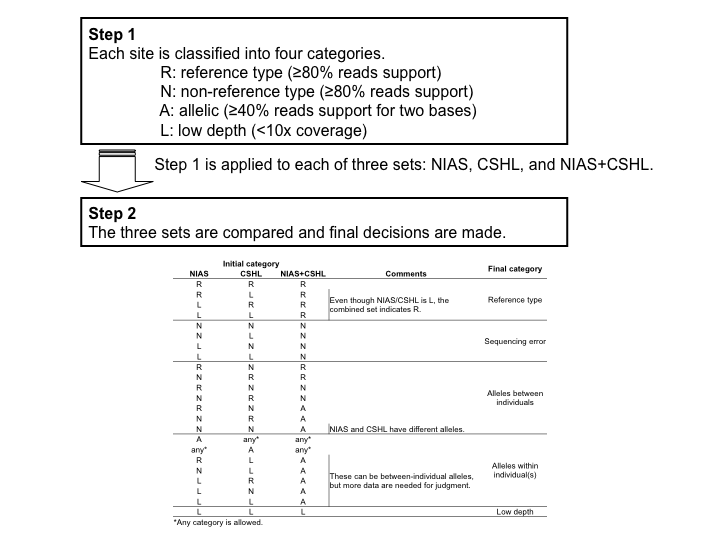

Supplement: Supplementary file 7 — Additional file 7:Figure S2. Classification of each site in the error corrections/SNP detections by Illumina reads. (TIFF 1 MB) [file 12284_2012_41_MOESM7_ESM.tiff]

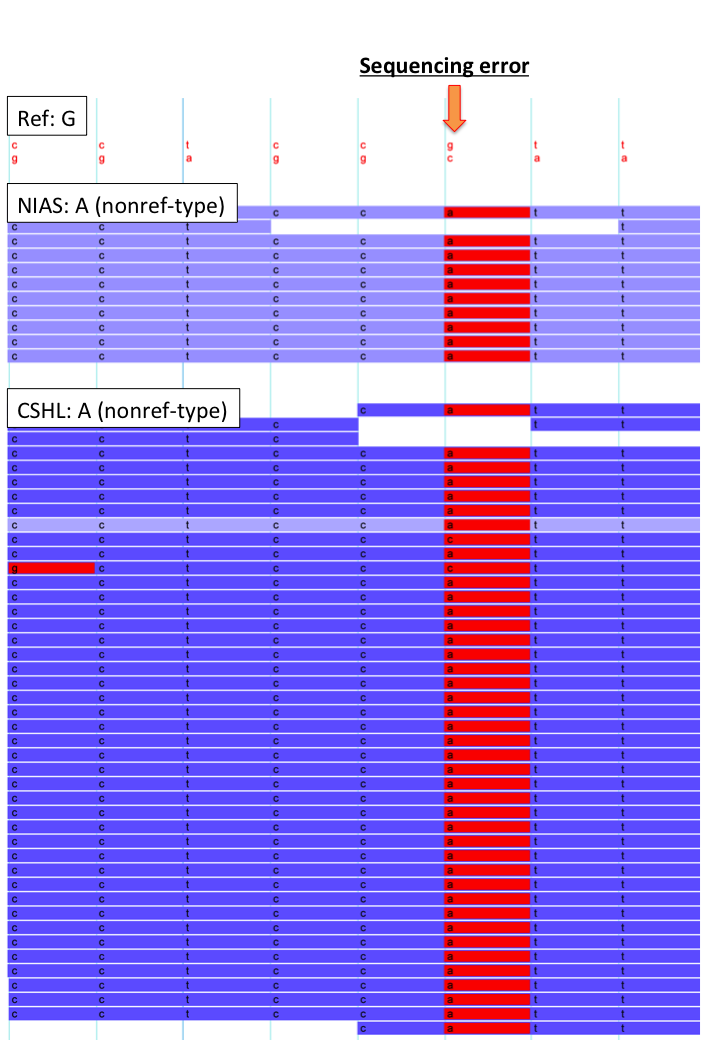

Supplement: Supplementary file 9 — Additional file 9:Figure S3. An example case of “sequencing error”. (TIFF 3 MB) [file 12284_2012_41_MOESM9_ESM.tiff]

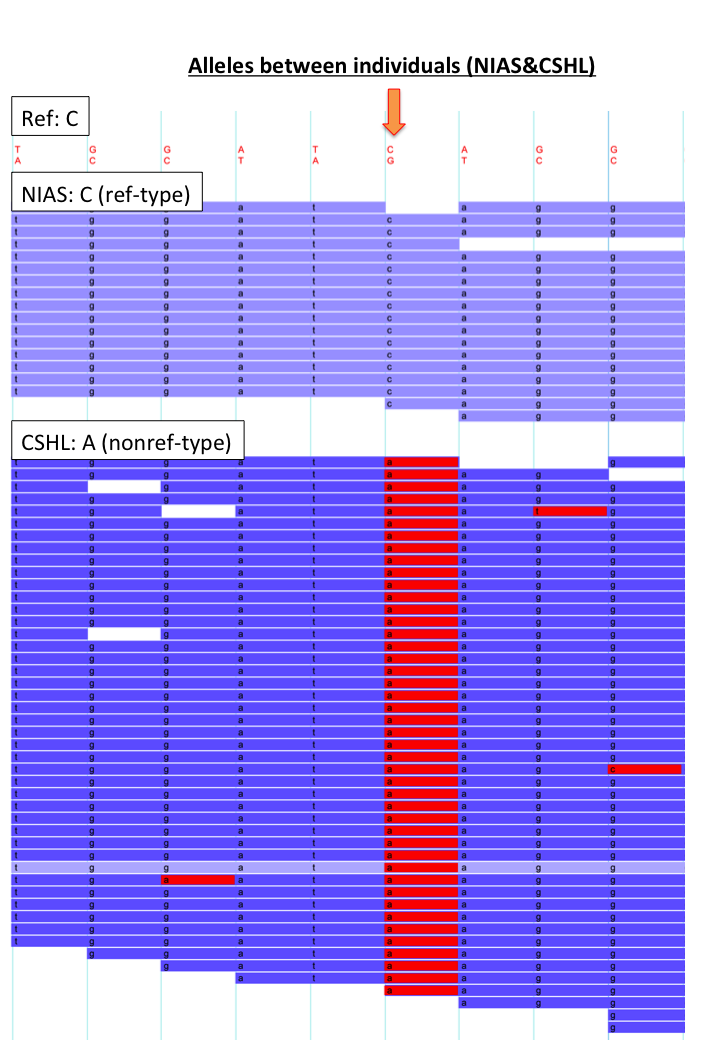

Supplement: Supplementary file 10 — Additional file 10:Figure S4. An example case of “alleles between individuals (NIAS and CSHL). (TIFF 3 MB) [file 12284_2012_41_MOESM10_ESM.tiff]

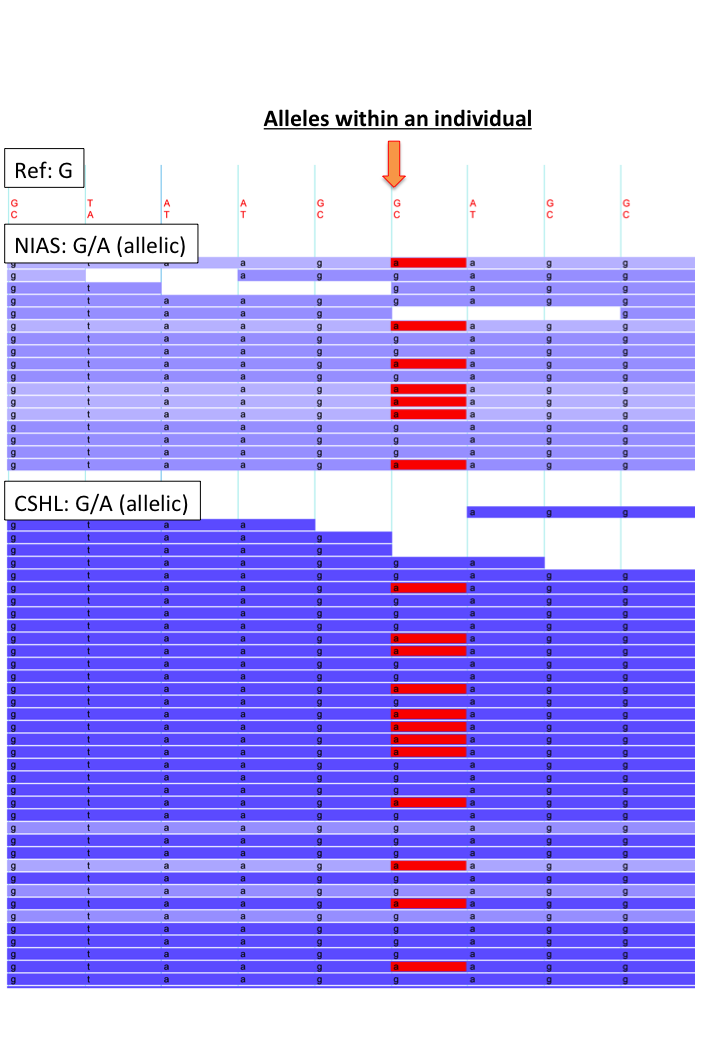

Supplement: Supplementary file 11 — Additional file 11:Figure S5. An example case of “alleles within individuals”. (TIFF 3 MB) [file 12284_2012_41_MOESM11_ESM.tiff]

**A**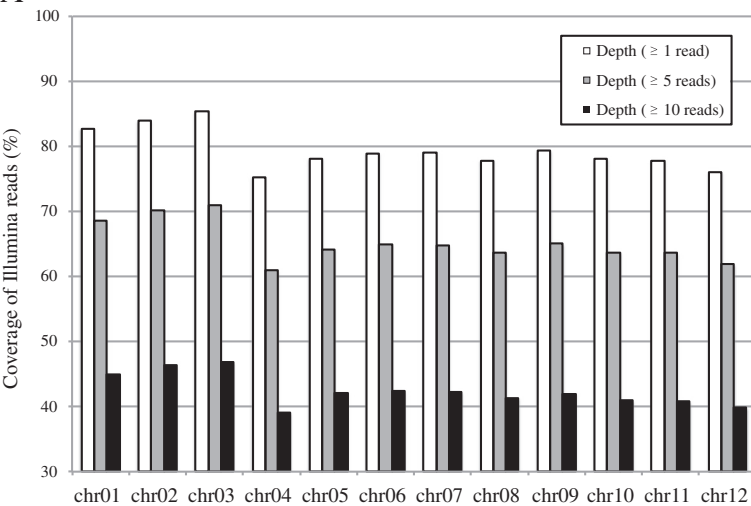**B**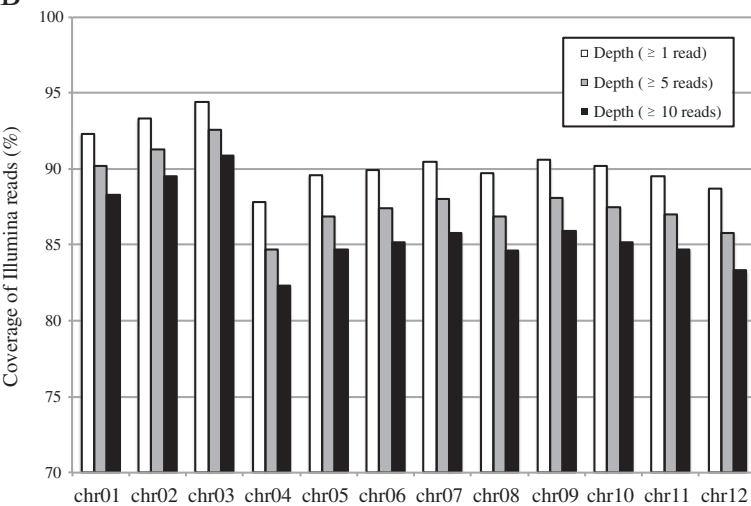**C**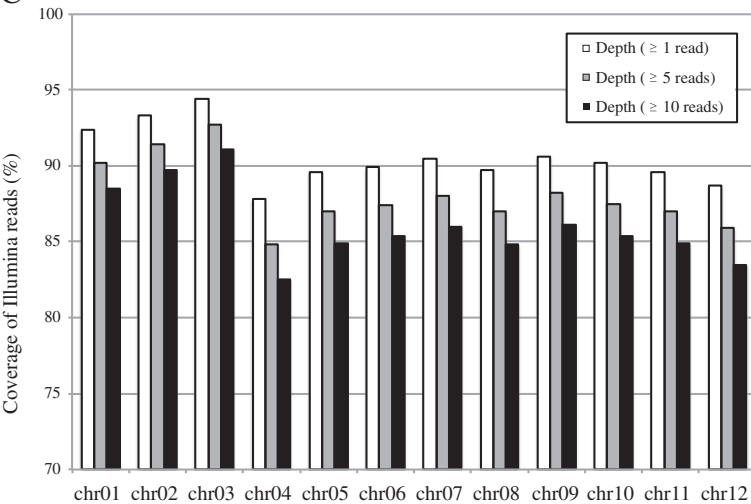

Supplement: Supplementary file 13 — Authors’ original file for figure 1 [file 12284_2012_41_MOESM13_ESM.pdf]

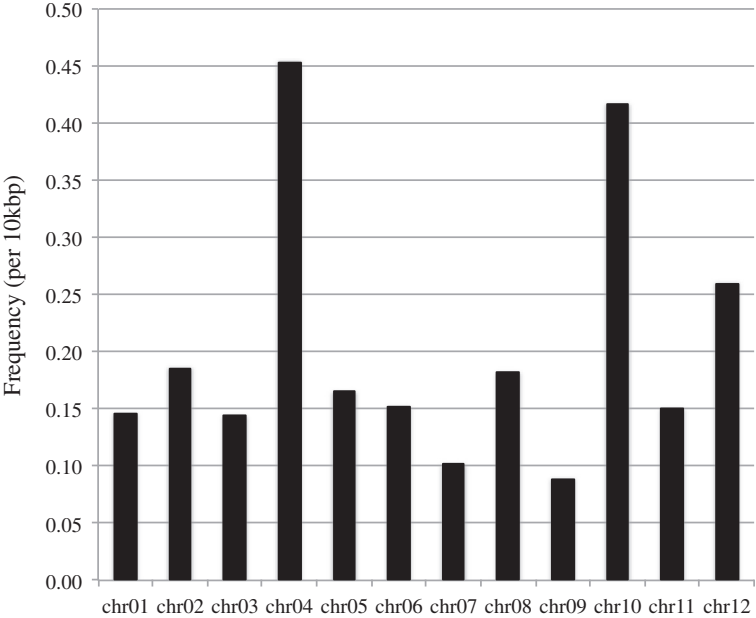

Supplement: Supplementary file 14 — Authors’ original file for figure 2 [file 12284_2012_41_MOESM14_ESM.pdf]
